# IFNγ induces PD-L1 overexpression by JAK2/STAT1/IRF-1 signaling in EBV-positive gastric carcinoma

**DOI:** 10.1038/s41598-017-18132-0

**Published:** 2017-12-19

**Authors:** Ji Wook Moon, Su-Kang Kong, Byung Soo Kim, Hyun Ji Kim, Hyangsoon Lim, Kyeonga Noh, Younghye Kim, Jung-Woo Choi, Ju-Han Lee, Young-Sik Kim

**Affiliations:** 1 Department of Pathology, Korea University College of Medicine, Seoul, Republic of Korea; 2 Department of Pathology, Korea University Ansan Hospital, Ansan, Republic of Korea

## Abstract

Programmed death-ligand 1 (PD-L1) acts as an immune checkpoint inhibitor in various cancers. PD-L1 is known to be more frequently expressed in EBV (+) gastric cancer (GC). However, the mechanisms underlying the regulation of PD-L1 expression in EBV (+) GC remain unclear. We investigated the basal and inducible PD-L1 expressions in GC cells. PD-L1 expression was upregulated upon treatment with IFNγ in both EBV (−) and EBV (+) GC cells. Upon stimulation with the same concentration of IFNγ for 24 h, EBV (+) SNU-719 cells showed dramatically higher PD-L1 expression levels by activating JAK2/STAT1/IRF-1 signaling than those of EBV (−) AGS cells. *PD-L1* promoter assays, chromatin immunoprecipitation, and electrophoretic mobility shift assays revealed that IFNγ-inducible PD-L1 overexpression is primarily mediated by the putative IRF-1α site of the *PD-L1* promoter in EBV (+) SNU-719 cells. Moreover, EBNA1 knockdown reduced both constitutive and IFNγ-inducible *PD-L1* promoter activity by decreasing the transcript and protein levels of JAK2 and subsequently STAT1/IRF-1/PD-L1 signaling. EBNA1 is suggested to be moderately enhance both constitutive and IFNγ-inducible PD-L1 expression in EBV (+) GC cells. Thus, the signaling proteins and EBNA1 that regulate PD-L1 expression are potential therapeutic targets in EBV (+) GC.

## Introduction

Programmed death ligand-1 (PD-L1), known as B7-H1 or CD274, is a glycoprotein of the B7 superfamily that is expressed on the cell surface of various tumor cells, as well as in lymphocytes, macrophages, and dendritic cells. PD-L1 functions as a PD-1 ligand, which binds to PD-1 on the cytotoxic T lymphocytes to inhibit the immune responses^1^. PD-L1 is upregulated in various epithelial and lymphoid tumors, including gastric cancer (GC)^2–5^. Anti-PD-L1 or anti-PD-1 antibody has recently been used as an immune checkpoint inhibitor^6^. Although the functional relationships between PD-L1 in cancer cells and PD-1 in cytotoxic T cells have been established^1,7–9^, the mechanisms by which PD-L1 expression is regulated in cancer cells remains unclear.

GCs have recently been categorized in The Cancer Genome Atlas (TCGA) as Epstein-Barr virus (EBV)-positive, microsatellite instable (MSI), chromosomal instable (CIN), and genomically stable (GS)^10^. EBV (+) GC accounts for about 10% of all GCs and PD-L1 overexpression is observed in over 50% of EBV (+) GCs. The prognostic significance of PD-L1 overexpression in GC patients remains controversial and has not yet been clearly defined^11–13^. Amplification of the 9p24.1 locus, which leads to PD-L1 overexpression, is observed in approximately 11–15% of EBV (+) GC cases^10,13^. However, other mechanisms responsible for PD-L1 upregulation aside from *PD-L1* gene amplification in EBV (+) GC have not been explored.

All EBV (+) GCs (EBV latency I or II) express EBV nuclear antigen 1 (EBNA1), EBERs, BARTs, and BART miRNAs. Approximately 50% of EBV (+) GC cases express LMP2A^14,15^. EBNA1 is a transcriptional factor in viral DNA replication and maintains the constant copy number of EBV genomes during cell division^16^. EBNA1 regulates the expression of other EBV genes and host cellular genes^17^. EBNA1 has a *cis*-acting immune evasive mechanism. The glycine-alanine repeat domain (GAr) in EBNA1 inhibits the translation of its own mRNA, thereby minimizing the production of antigenic peptides that activate the major histocompatibility complex (MHC) class I pathway^18^. Moreover, EBNA1 has been shown to increase STAT1 expression in three different carcinoma cell lines^19,20^. EBNA1 enhances phosphorylation and nuclear localization of STAT1 in response to IFNγ^20^.

IFNγ is a cytokine secreted by tumor–infiltrating T cells and induce PD-L1 expression by stimulating the JAK/STAT signaling pathway in myeloid leukemia cells^8^. IFNγ also regulates IRF-1 expression in lung cancer cells^7^. However, the roles of IFNγ and EBNA1 in PD-L1 expression of EBV (+) GC remain to be determined.

Therefore, we investigated whether IFNγ induces PD-L1 expression and compared the mechanisms by which EBV influences IFNγ-induced PD-L1 expression in EBV (+) GC and EBV (−) GC cells.

## Results

### Constitutive PD-L1 expression correlates with EBNA1 expression in EBV (+) GC

**Figure 1:**
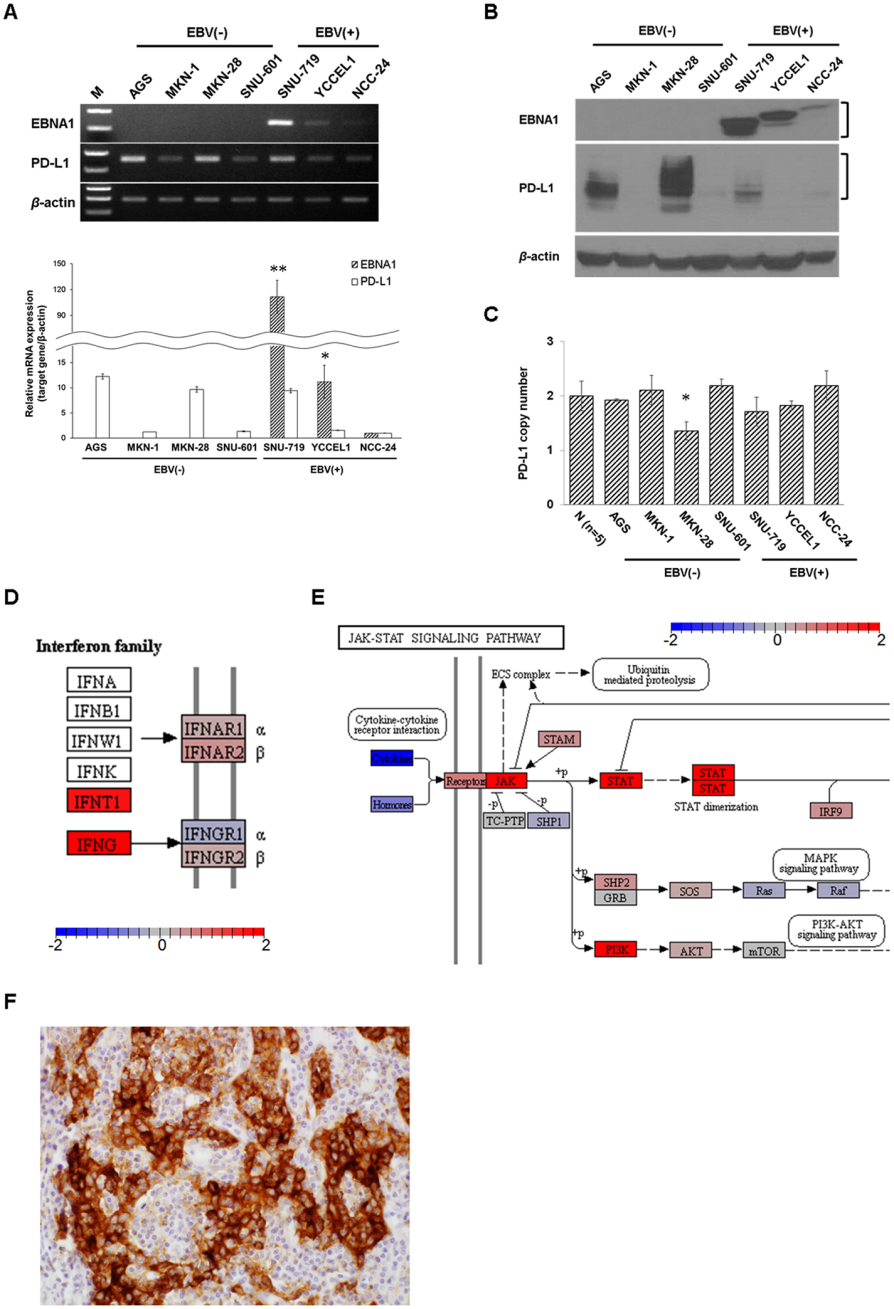
Constitutive PD-L1 expression correlates with EBNA1 expression and inducible PD-L1 expression is suggested to be mediated by IFNγ/JAK/STAT pathway activation in EBV (+) GC. (**A**) Relative mRNA expression levels of EBNA1 and PD-L1 in seven GC cell lines were determined by RT-PCR (upper panel) and qRT-PCR (lower panel). The data are presented as mean ± SEM (n = 3). *P < 0.05 and **P < 0.001 compared to EBNA1 mRNA levels of NCC-24 cell line (Student’s t-test). M, 100 bp DNA ladder. (**B**) Protein expression of EBNA1 and PD-L1 in seven GC cell lines were determined by immunoblot analysis. (**C**) Copy numbers of the *PD-L1* gene were determined by qRT-PCR in seven GC cell lines. The average copy number of five normal gastric tissue samples was used as a normal control (N). RNase P was used as loading control. The data are presented as mean ± SEM (n = 3). *P < 0.05 compared to normal control (Student’s t-test). (**D**) Gene set enrichment analysis (GSEA) of TCGA EBV (+) GC data was performed using the ensemble of GSEA bioconductor R package. Gene expression fold changes were projected onto the Kyoto Encyclopedia of Genes and Genomes (KEGG) pathway maps. Differential expression of interferon family and related receptor genes (http://www.genome.jp/kegg-bin/show_pathway?hsa04060). (**E**) Differential expression of JAK/STAT or PI3K signaling pathway genes between EBV (+) GC and CIN–type GC tissues (http://www.genome.jp/kegg-bin/show_pathway?hsa04630). Upregulated and downregulated expression levels are colored in red and blue, respectively. (**F**) Immunohistochemical analysis of PD-L1 in EBV (+) GC tissues revealed homogeneous and high PD-L1 expression in GC cells contacted by infiltrating lymphocytes. *β*-actin was used as a loading control.

We examined the constitutive expression of PD-L1 in four EBV (−) cell lines, namely, AGS, MKN-1, MKN-28, and SNU-601, and in three EBV (+) cell lines, namely, SNU-719, YCCEL1, and NCC-24. PD-L1 expression was compared with that of EBNA1 in three EBV (+) GC cell lines. PD-L1 was constitutively overexpressed in both mRNA and protein levels in the EBV (−) AGS and MKN-28 cell lines, as well as in the EBV (+) SNU-719 cell line (Fig. 1A,B). SNU-719 showed the highest EBNA1 expression levels, followed by YCCEL1 and NCC-24. Interestingly, PD-L1 expression levels were proportional to EBNA1 levels (Fig. 1A,B). Previous reports have indicated that PD-L1 overexpression is associated with amplification of the *PD-L1* gene in about 11–15% of EBV (+) GCs^10,13^. By contrast, in this study, all GC cell lines, except for MKN-28, had normal copy numbers of the *PD-L1* gene; however, AGS and SNU-719 cells showed high PD-L1 levels (Fig. 1C). Paradoxically, MKN-28 cells had relatively low in *PD-L1* gene copy numbers, but had high PD-L1 protein levels (Fig. 1B,C). These results suggest that GC cells can express PD-L1 proteins through a mechanism different from *PD-L1* gene amplification.

Considering the bioinformatics analysis that IFNγ/JAK/STAT pathway is overexpressed in EBV (+) GCs (Fig. 1E), we examined basal expression of JAK2/STAT1/IRF-1 signaling proteins in seven GC cell lines. In the basal state, AGS and SNU-719 cells expressed JAK2/STAT1/IRF-1 signaling proteins. In detail, JAK2 was the most expressed in SNU-601 and SNU-719 and higher in AGS, MKN-1, and MKN28 than YCCEL1 and NCC-24. STAT1 expression was highest in MKN-28, followed by SNU719, YCCEL1 and NCC-24. IRF-1 was more expressed in AGS, MKN-1, and NCC-24 than MKN-28 and SNU-719 cells (Supplementary Fig. S1A). When IFNγ is released from cytotoxic T cells, IFNγ binding to its receptor results in rapid and dramatic increased formation of the IFNγ receptor (IFNGR) heterotetrameric complex, which consists of IFNGR1 and IFNGR2, on the tumor cell membranes and subsequently initiates the JAK/STAT/IRF-1 signaling pathway^21,22^. All GC cell lines constitutively expressed IFNGR2, JAK2, STAT1, IRF-1, and PD-L1 mRNAs at varying levels, but IFNGR1 expression was relatively low or absent compared to IFNGR2 (Supplementary Fig. S1B). Among these, AGS and SNU-719 cell lines that showed high PD-L1 expression levels were selected for further experiments.

### IFNγ induces higher PD-L1 expression in EBV (+) GC compared to EBV (−) GC

Differentially expressed interferon family proteins and related signaling proteins were defined based on GSEA using the TCGA RNA-sequencing data. Comparison of ten EBV (+) GC and five CIN–type GC tissues showed that IFNγ and IFNT1 were significantly enriched in EBV (+) GC cases than in CIN–type GC (Fig. 1D). In addition, the JAK/STAT and PI3K signaling pathways were highly enriched in EBV (+) GC (Fig. 1E). These results suggest the involvement of IFNγ in the regulation of the JAK/STAT signaling pathway in EBV (+) GC, consistent with previous evidence showing that IFNγ activates JAK/STAT signaling in hematopoietic cells^8^ and induces PD-L1 expression through JAK/STAT signaling in lung cancer cells^7^. Furthermore, immunohistochemical analysis of EBV (+) GC tissues showed that PD-L1 was particularly strongly expressed in EBV (+) GC cells confronting infiltrating lymphocytes (Fig. 1F). Therefore, we hypothesized that IFNγ released from cytotoxic T lymphocytes could induce high levels of PD-L1 expression in EBV (+) GC cells.

**Figure 2:**
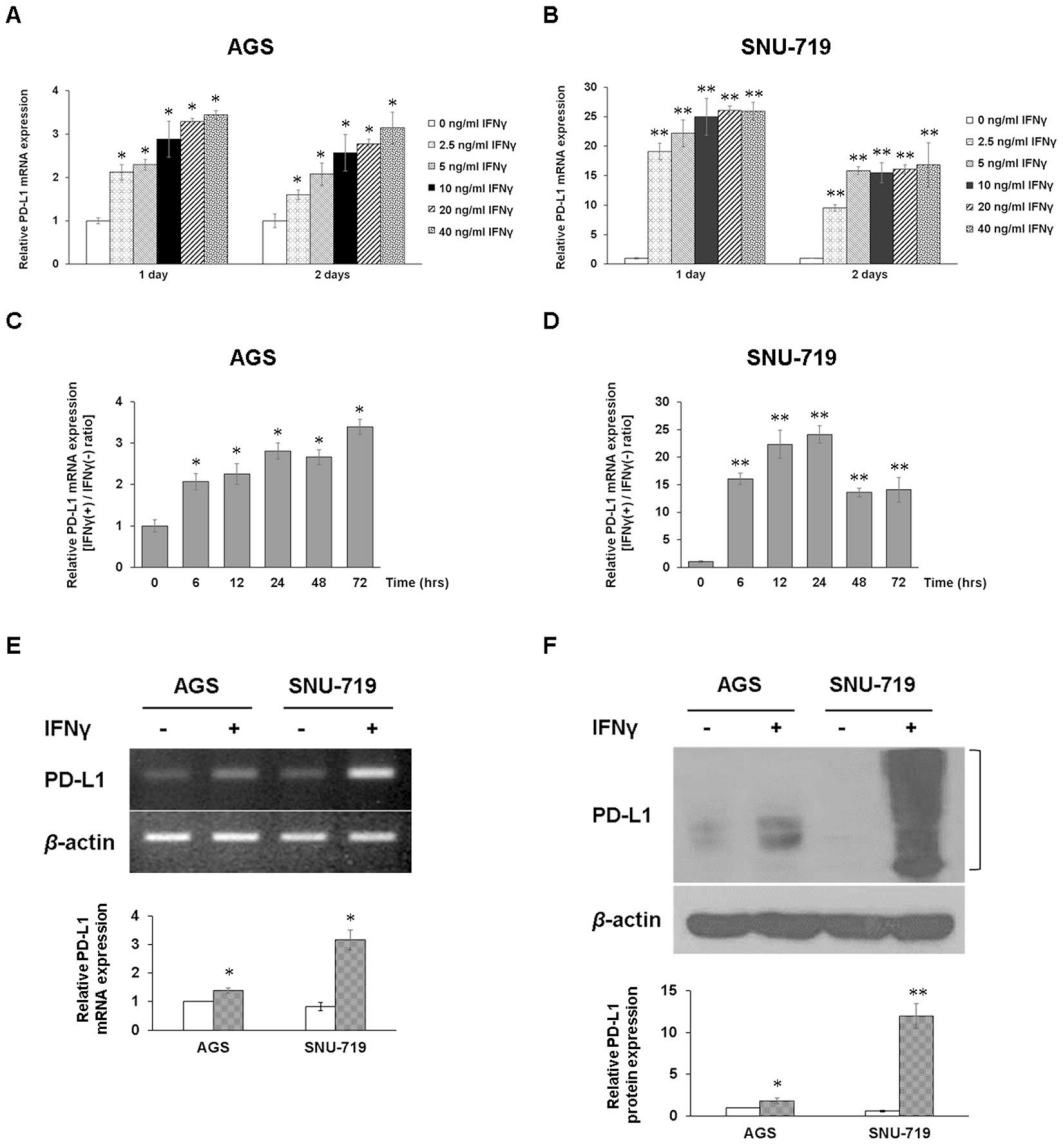
IFNγ induces significantly higher PD-L1 mRNA and protein expression in EBV (+) GC than in EBV (−) GC. IFNγ treatment increases PD-L1 levels in a dose- and time-dependent manner. (**A**,**B)** After stimulating EBV (−) AGS cells and EBV (+) SNU-719 with increasing doses of IFNγ for 24 and 48 h, PD-L1 expression levels were determined by qRT-PCR. The data are presented as mean ± SEM (n = 3). *P < 0.05 and **P < 0.001 compared to the same unstimulated cell line (Student’s t-test). (**C**,**D)** After treating EBV (−) AGS cells and EBV (+) SNU-719 with 10 ng/mL IFNγ for the indicated time periods, PD-L1 expression levels were determined by qRT-PCR. The data are presented as mean ± SEM (n = 3). *P < 0.05 and **P < 0.001 compared to the same unstimulated cell line (Student’s t-test). (**E**,**F)** After treatment of AGS and SNU-719 cell lines with 10 ng/mL IFNγ for 24 h, relative PD-L1 mRNA and protein expression levels were determined by RT-PCR and immunoblot analyses. The mRNA and protein levels of PD-L1 were quantified relative to β-actin. The data are presented as mean ± SEM (n = 3). *P < 0.05 and **P < 0.001 compared to the same unstimulated cell line (Student’s t-test).

To compare the expression of PD-L1 by IFNγ in EBV (−) and EBV (+) GC cells, the appropriate concentration and duration of IFNγ treatments was first determined. PD-L1 expression in GC cells under the optimal IFNγ treatment conditions was then assayed (Fig. 2). PD-L1 mRNA levels were augmented by gradually increasing IFNγ stimulation in AGS and SNU-719 cells. PD-L1 mRNA levels peaked at 40 ng/mL upon IFNγ stimulation for 24 h, showing 3.4-fold and 26-fold increases in AGS and SNU-719 cells, respectively (Fig. 2A,B). The stimulatory effect of IFNγ on PD-L1 expression was dose-dependent and the optimal concentration was 10 ng/mL. When AGS cells were stimulated with IFNγ at 10 ng/mL, the level of PD-L1 mRNA was continuously increased by 2.8-fold up to 24 h and maximal expression was observed at 72 h of stimulation (Fig. 2C). In contrast, when stimulated with the same dose of IFNγ in SNU-719 cells, the level of PD-L1 mRNA reached a peak 24-fold at 24 h and subsequently decreased at 48 h and 72 h after stimulation with IFNγ (Fig. 2D). It was noteworthy that when GC cells were stimulated with 10 ng/mL of IFNγ for 24 h, EBV (+) SNU-719 cells showed significantly higher PD-L1 mRNA and protein levels than those of EBV (−) AGS cells (Fig. 2E,F). Furthermore, EBV (+) YCCEL and NCC-24 cells also showed that IFNγ induced significantly higher PD-L1 expression compared to EBV (−) AGS cells, similar to SNU-719 cells. The inducible PD-L1 expression was correlated with EBNA1 expression in the three EBV (+) cell lines (Supplementary Fig. S2).

### IFNγ-induced PD-L1 expression is mediated by JAK2/STAT1/IRF-1 signaling in EBV (+) GC

**Figure 3:**
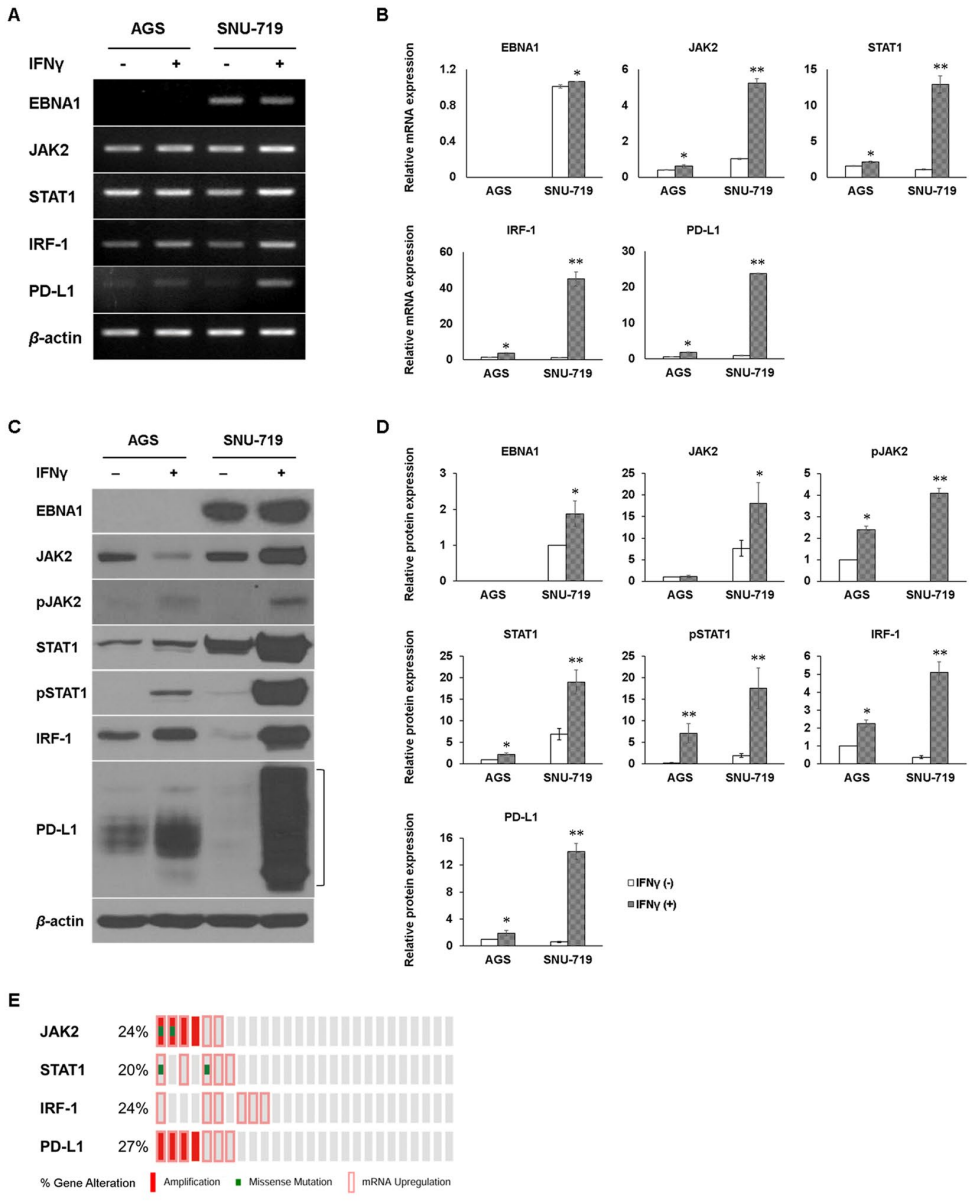
IFNγ treatment induces PD-L1 upregulation via the activation of JAK2/STAT1/IRF-1 signaling in EBV (+) GC cells. (**A**,**B**) After AGS and SNU-719 cell lines were stimulated with 10 ng/mL IFNγ for 24 h, relative mRNA levels of EBNA1, JAK2, STAT1, IRF-1 and PD-L1 were determined by RT-PCR and qRT-PCR, respectively. *β*-actin was used as a loading control. The mRNA levels by qRT-PCR were quantified and compared with those of the same unstimulated cells. The data are presented as mean ± SEM (n = 3). *P < 0.05 and **P < 0.001 compared to the same unstimulated cell line (Student’s *t*-test). (**C**,**D)** The protein levels of EBNA1, JAK2, pJAK2, STAT1, pSTAT1, IRF-1, and PD-L1 were quantified relative to β-actin. The data are presented as mean ± SEM (n = 3). *P < 0.05 and **P < 0.001 compared to the same unstimulated cell line (Student’s *t*-test). (**E)** JAK2/STAT1/IRF-1/PD-L1 signaling was also analyzed by bioinformatic OncoPrint using TCGA EBV (+) GC data.

Previous studies^7,8,21^ and our current GSEA results suggest that IFNγ induces PD-L1 expression via activation of JAK2/STAT1/IRF-1 signaling in EBV (+) GC cells. To verify these results, EBNA1, PD-L1, and JAK2/STAT1/IRF-1 signaling pathway were examined by RT-PCR, qRT-PCR, and immunoblotting. Alteration of these genes were also analyzed using the OncoPrint from TCGA EBV (+) GC tissue data. IFNγ induced PD-L1 upregulation at both the mRNA and protein levels via phosphorylation of JAK2 and STAT1 and subsequent increase of IRF-1 expression in EBV (+) SNU-719 cells (Fig. 3A,B,C, and D). Surprisingly, IFNγ-induced PD-L1 expression was considerably higher in EBV (+) SNU-719 cells than in EBV (−) AGS cells. Notably, IFNγ treatment increased EBNA1 and total JAK2 expression levels in SNU-719 cells (Fig. 3C,D). These data suggest that EBNA1 co-operates with IFNγ and increases the transcriptional expression of JAK2, leading to IFNγ-induced PD-L1 overexpression.

Additionally, OncoPrint results indicate that JAK2, STAT1, IRF-1, and PD-L1 genes were altered or overexpressed in about 24%, 20%, 24%, and 27% of EBV (+) GC tissues, respectively. All EBV (+) GC tissues showed almost the same gene alterations or transcriptional upregulation patterns. Interestingly, this phenomenon was also observed in the EBV (+) GC cases without *JAK2* or *PD-L1* gene amplification (Fig. 3E).

**Figure 4:**
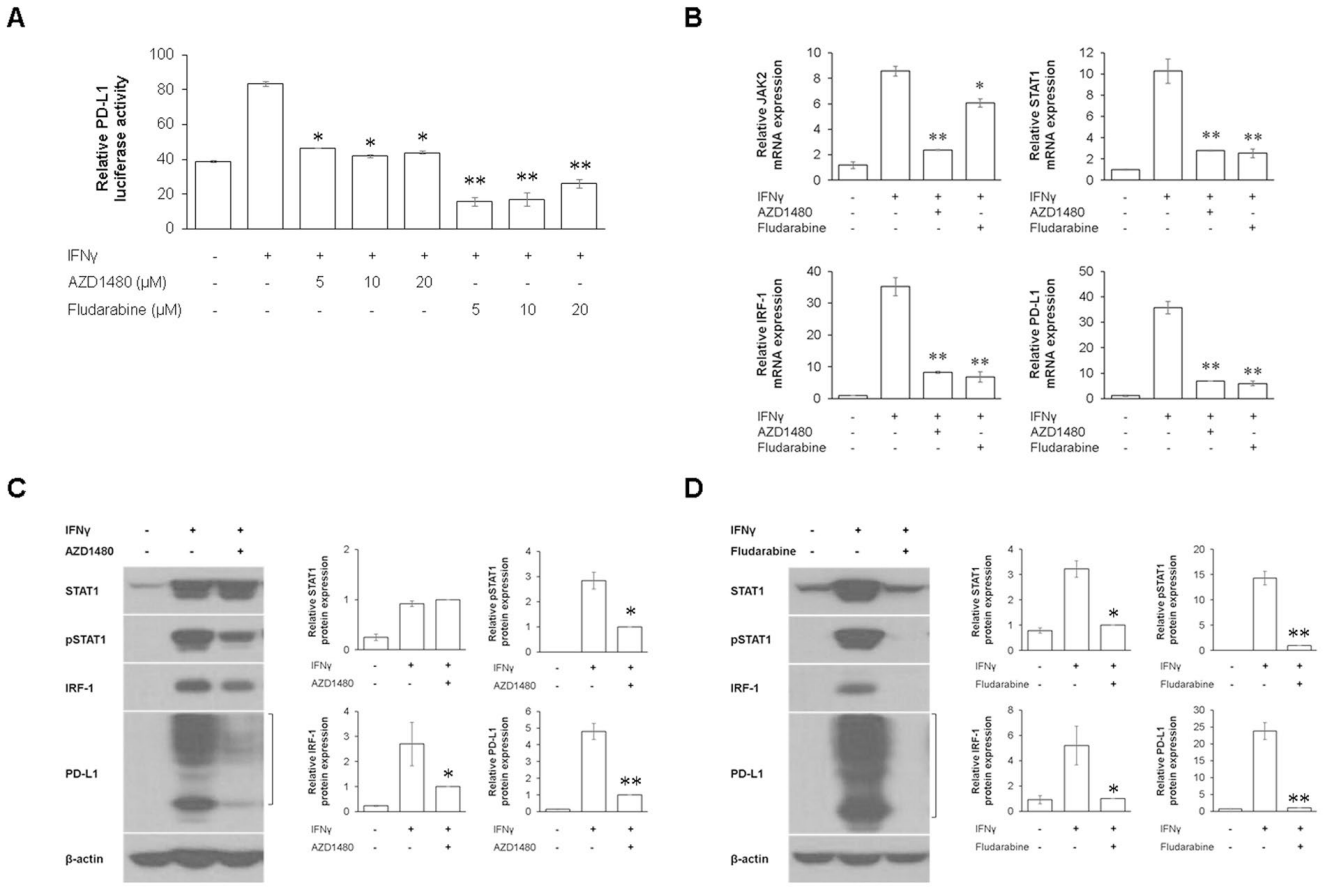
IFNγ-induced JAK2/STAT1/IRF-1/PD-L1 signaling is inhibited with AZD1480 (an ATP-competitive JAK2 inhibitor) and fludarabine (a STAT1 inhibitor depleting STAT1 mRNA and protein) in EBV (+) GC cells. (**A**) EBV (+) SNU-719 cells were incubated with increasing concentrations of AZD1480 or fludarabine (5, 10, and 20 μM) for 2 h, and then transfected with the PD-L1 promoter luciferase vector. After 4 h, cells were stimulated with 10 ng/mL IFNγ for 24 h, luciferase activities were measured. (**B**) EBV (+) SNU-719 cells were treated with 5 μM of AZD1480 or fludarabine for 2 h, followed by stimulation with 10 ng/mL IFNγ for 24 h. The mRNA levels of JAK2, STAT1, IRF-1, and PD-L1 were determined by qRT-PCR. (**C**, **D**) After treatments with AZD1480 or fludarabine under the same conditions as (**B**), the protein levels of STAT1, IRF-1, and PD-L1 proteins were determined by immunoblotting and quantified relative to β-actin. The data are presented as mean ± SEM (n = 3). *P < 0.05 and **P < 0.001 compared to only IFNγ-stimulated SNU-719 cell line (Student’s *t*-test).

To determine whether IFNγ-induced PD-L1 overexpression is mediated by activation of JAK2/STAT1/IRF-1 signaling, we investigated PD-L1 promoter activities and expression changes of JAK2, STAT1, IRF-1, and PD-L1 in IFNγ-stimulated SNU-719 cells after treatments with AZD1480 (an ATP-competitive JAK2 inhibitor) and fludarabine (a STAT1 inhibitor that depletes STAT1 mRNA and protein). The luciferase activities of *PD-L1* promoter were significantly decreased in IFNγ-stimulated SNU-719 cells pretreated with AZD1480 or fludarabine, compared to only IFNγ-stimulated SNU-719 cells (Fig. 4A). The JAK2 inhibitor, AZD1480 blocked IFNγ-induced increase of JAK2, STAT1, IRF-1, and PD-L1 mRNAs. The STAT1 inhibitor, fludarabine significantly inhibited IFNγ-induced upregulation of STAT1, IRF-1, and PD-L1 mRNAs with a moderate suppression of JAK2 mRNA upregulation (Fig. 4B). AZD1480 decreased the protein levels of IRF-1 and PD-L1 expression via the inhibition of STAT1 phosphorylation (Fig. 4C), whereas fludarabine significantly reduced the protein levels of IRF-1 and PD-L1 expression via the inhibition of STAT1 expression and phosphorylation (Fig. 4D). These results indicate that IFNγ-induced PD-L1 expression is mediated by JAK2/STAT1/IRF-1 signaling in EBV (+) GC cells.

### IFNγ-induced IRF-1 primarily binds to the IRF-1α binding site of *PD-L1* promoter in EBV (+) GC

**Figure 5:**
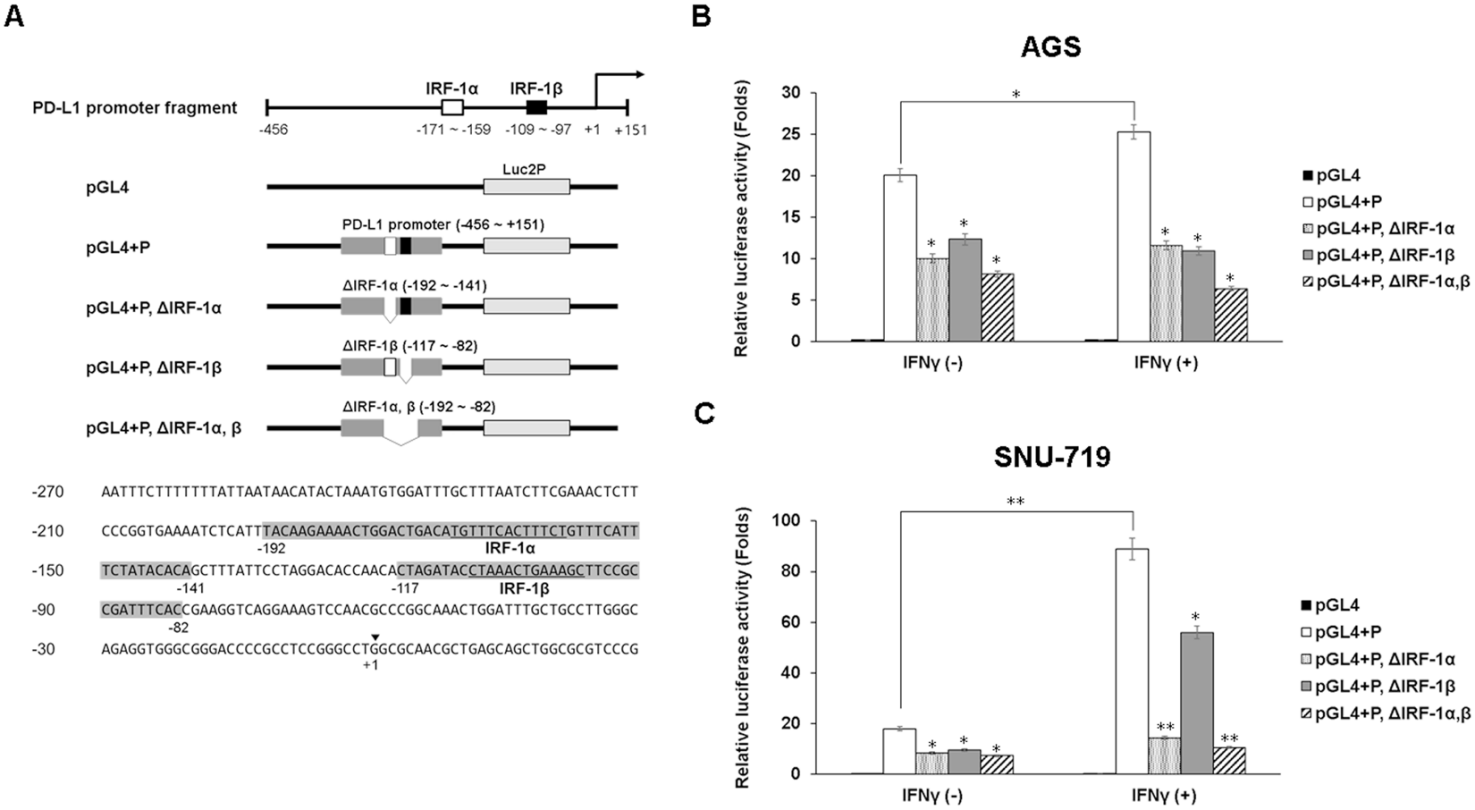
IFNγ augments *PD-L1* promoter activity by binding to the putative IRF-1α sequence (−171 to −159) in EBV (+) GC cells. (**A)** Schematic representation of the promoter deletion constructs and nucleotide sequence of the *PD-L1* promoter. The 456-bp sequence of the 5′-flanking region of *PD-L1* is shown. The transcription start site is indicated by +1. Underlined sequences represent two putative IRF-1 binding sites (IRF-1α and -1β). Shaded sequences are deleted for the promoter constructs. **(B**,**C)** EBV (−) AGS and EBV (+) SNU-719 cell lines were transfected with the reporter constructs. After 4 h, cells were stimulated with 10 ng/mL IFNγ for an additional 24 h, luciferase activities were measured. The data are presented as mean ± SEM (n = 3). *P < 0.05 and **P < 0.001 compared to the same unstimulated cell line (Student’s t-test).

To identify the promoter regions of *PD-L1* that are regulated by IFNγ and EBV, we measured *PD-L1* promoter activity using the reporter constructs in EBV (−) and EBV (+) IFNγ–stimulated and unstimulated GC cells (Fig. 5). A fragment (−456 to +151) of the *PD-L1* promoter contains two putative IRF-1 binding sites, designated as IRF-1α and IRF-1β^7^. The 600-bp region of the *PD-L1* promoter was amplified from human genomic DNA by PCR and cloned into a luciferase reporter plasmid, the pGL4.15 basic vector (Fig. 5A). Luciferase activity of the *PD-L1* promoter (pGL4 + P, −456 to +151) was induced 1.3 times higher in IFNγ-treated EBV (−) AGS cells than in the untreated control (Fig. 5B). Surprisingly, however, *PD-L1* promoter luciferase activity was fivefold higher in IFNγ-stimulated EBV (+) SNU-719 cells (Fig. 5C). Luciferase activities of the IRF-1α-, IRF-1β-, and IRF-1α,β-deleted constructs in unstimulated AGS cells decreased by 47%, 39%, and 56%, respectively. Promoter activities in IFNγ-stimulated AGS cells showed no significant differences compared to those of unstimulated AGS cells (Fig. 5B). As with AGS cells, luciferase activities of these constructs in unstimulated SNU-719 cells were reduced by 52%, 41%, and 53%, respectively. Interestingly, the luciferase activities of both the IRF-1α- and IRF-1α, β-deleted constructs were significantly reduced by 84% and 88%, respectively, in IFNγ-stimulated SNU-719 cells. The promoter activity of the IRF-1β-deleted construct in IFNγ-stimulated SNU-719 cells decreased by 37%, similar to the 41% decrease of the promoter activity in unstimulated SNU-719 cells (Fig. 5C). These results imply that basal and inducible PD-L1 expressions are mediated through the comparably equal binding of IRF-1 to both IRF-1α and IRF-1β nucleotide sites in IFNγ-stimulated and unstimulated EBV (−) GC cells, as well as unstimulated EBV (+) GC cells. In contrast, the transcriptional PD-L1 overexpression in IFNγ-stimulated EBV (+) GC cells is mediated by signaling through the IRF-1α binding site rather than the IRF-1β. Therefore, EBV is suggested to play a role in IFNγ-induced PD-L1 overexpression.

**Figure 6:**
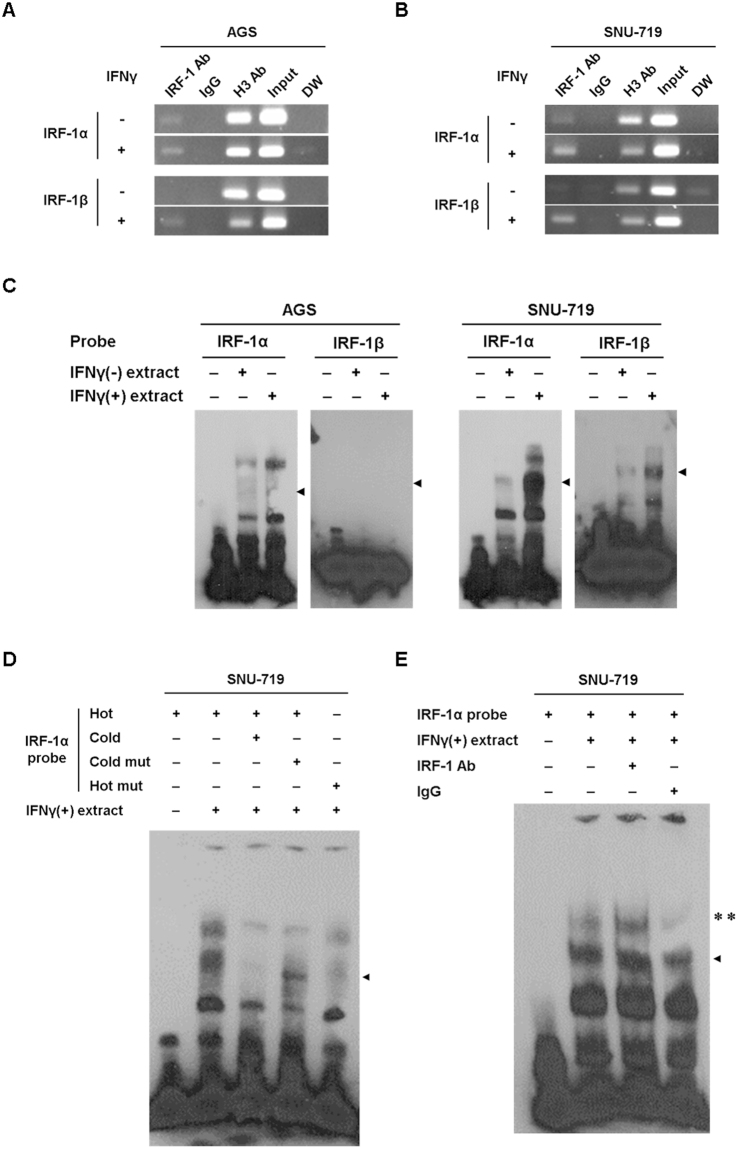
IFNγ-stimulated IRF-1 binds to the IRF-1α sequence of the *PD-L1* promoter in EBV (+) GC. (**A**,**B)** Chromatin immunoprecipitation (ChIP) assays were performed on IFNγ-stimulated or unstimulated EBV (−) and EBV (+) GC cells using anti-IRF-1 antibody and sequence-specific primers (IRF-1α and IRF-1β) targeting the IRF-1 binding regions of the *PD-L1* promoter. After AGS and SNU-719 cells were stimulated with 10 ng/mL IFNγ for 24 h, immunoprecipitated DNA-protein complexes were analyzed by PCR and normalized against input DNA. Anti-H3 antibody and IgG were used as positive and negative controls, respectively. DW, distilled water. (**C)** Electrophoretic mobility shift assay (EMSA) was performed using nuclear extracts from IFNγ-stimulated or unstimulated AGS and SNU-719 GC cells and biotin-labeled double-strand oligonucleotide probes (IRF-1α and IRF-1β). (**D)** Competitive EMSA for evaluating the specificity of the IRF-1α binding site was performed on IFNγ-stimulated SNU-719 cells using the biotin-labeled wild-type (Hot), unlabeled wild-type (Cold), unlabeled mutant (Cold mut), and biotin-labeled mutant (Hot mut) IRF-1α probes. (**E)** Supershift assays for evaluating the binding of IRF-1 at the IRF-1α site were performed using the wild-type IRF-1α probe, IFNγ-stimulated SNU-719 nuclear extracts, and anti-IRF-1 antibody. IgG was used as negative control. The double asterisk and arrowheads indicate a supershifted band and DNA-protein complexes, respectively.

To verify whether IRF-1 binds to the putative IRF-1 binding sites within the human *PD-L1* promoter *in vivo*, we conducted ChIP assays in IFNγ-stimulated or unstimulated AGS and SNU-719 cells. In the basal states of AGS and SNU-719 cells, IRF-1 was specifically bound to the IRF-1α binding region, but not the IRF-1β region (Fig. 6A,B). By contrast, in both IFNγ-stimulated AGS and SNU-719 cells, specific bindings of IRF-1 at the IRF-1α and IRF-1β sites of the *PD-L1* promoter were found to significantly increase (Fig. 6A,B). Notably, IFNγ-induced IRF-1 showed considerably higher binding at the IRF-1α than at the IRF-1β site. Binding of IRF-1 to IRF-1α upon IFNγ treatment in SNU-719 cells was significantly higher, compared to that of the same *PD-L1* promoter site in AGS cells (Fig. 6A,B).

To test the binding of IRF-1 to the *PD-L1* promoter regions *in vitro*, we performed EMSA using the nuclear extracts from IFNγ-stimulated or unstimulated AGS and SNU-719 cells. GC cells were stimulated with 10 ng/mL of IFNγ for 24 h. IFNγ treatment increased protein-DNA complex formation between the nuclear proteins and the IRF-1α DNA probe in both AGS and SNU-719 cells (Fig. 6C). In particular, IFNγ significantly increased the formation of protein–IRF-1α oligonucleotide complex in SNU-719 cells than in AGS cells. However, the IRF-1β probe did not form any detectable complex in the basal and IFNγ-induction states of AGS cells.

To identify the specific IRF-1 binding at the IRF-1α site of the *PD-L1* promoter, we performed competition EMSA and supershift assays using IFNγ-stimulated SNU-719 cells. Protein-DNA complex formation by IFNγ-stimulated nuclear extracts and hot wild-type probes at the IRF-1α site were competitively inhibited, with over 50-fold excess of the unlabeled cold or hot mutant probes. Complex formation was restored by the cold mutant probes (Fig. 6D). Additionally, the shifted signal produced by the IRF-1-DNA complex was supershifted in the presence of anti-IRF-1 antibody but not IgG (Fig. 6E). These results provide evidence that IRF-1 is induced by IFNγ and specifically binds to the IRF-1α sequence in the *PD-L1* promoter in EBV (+) GC cells.

### EBNA1 knockdown decreases constitutive and IFNγ-mediated PD-L1 expression by downregulating JAK2 expression

**Figure 7:**
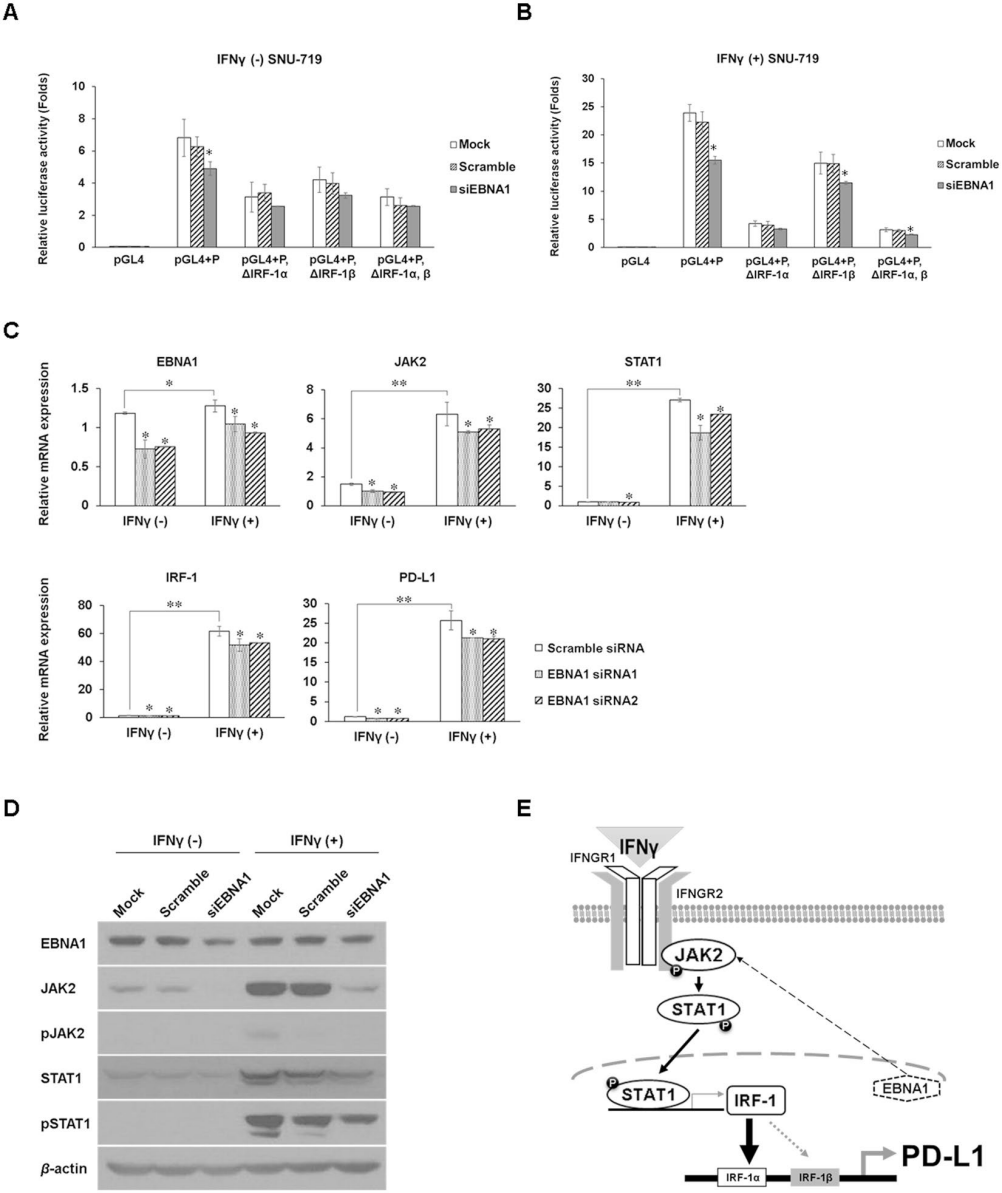
EBNA1 siRNA knockdown reduces constitutive and IFNγ-induced JAK2/STAT1/IRF-1/PD-L1 signaling and schematic working model in EBV (+) GC. (**A**) Following transfection with EBNA1 siRNA or scramble siRNA for 48 h, SNU-719 cells were transfected with the reporter constructs for 24 h, and luciferase activities were determined. The data are presented as mean ± SEM (n = 3). *P < 0.05 compared to the same unstimulated cell line (Student’s t-test). (**B**) After SNU-719 cells were stimulated with 10 ng/mL of IFNγ for 24 h and were transfected with EBNA1 siRNA or scramble siRNA for 48 h, the cells were transfected with the reporter constructs for 24 h and the luciferase activities were determined. The data are presented as mean ± SEM (n = 3). *P < 0.05 compared to the same unstimulated cell line (Student’s t-test). (**C**) SNU-719 cells were stimulated with 10 ng/mL IFNγ for 24 h and were transfected with EBNA1 siRNAs or scramble siRNA for 48 h. mRNA expression levels of EBNA1, JAK2, STAT1, IRF-1, and PD-L1 relative to unstimulated cells were quantified by qRT-PCR. The data are presented as mean ± SEM (n = 3). *P < 0.05 and **P < 0.001 compared to the same unstimulated cell line (Student’s t-test). (**D**) SNU-719 cells were stimulated with 10 ng/mL IFNγ for 24 h and subsequently transfected with EBNA1 siRNAs or scramble siRNA for 48 h. Protein expression levels of EBNA1, JAK2, pJAK, STAT1, and pSTAT1 were analyzed by immunoblotting compared to unstimulated SNU-719 cells. *β*-actin was used as a loading control. *P < 0.05 and **P < 0.001. (**E**) Following IFNγ stimulation, EBNA1 enhances PD-L1 expression by upregulating JAK2 expression and subsequently activating JAK2/STAT1/IRF-1 signaling in EBV (+) GC cells.

To examine whether the PD-L1 expression is transcriptionally regulated by EBNA1, we measured the *PD-L1* promoter activity in SNU-719 cells with or without IFNγ treatment after EBNA1 knockdown with siRNA. *PD-L1* full promoter activity was significantly decreased in SNU-719 cells with EBNA1 knockdown compared to mock and scramble siRNA-transfected control SNU-719 cells. However, activities of the three IRF-1-deleted promoters were not significantly decreased (Fig. 7A). By contrast, the EBNA1- knockdown SNU-719 cells were treated with IFNγ for 24 h, luciferase activities of the full and deleted *PD-L1* promoters, except for the IRF-1α-deleted promoter, were significantly decreased (Fig. 7B). These results indicate that EBNA1 induces basal PD-L1 expression through the IRF-1α and IRF-1β sequences, whereas it promotes IFNγ-induced PD-L1 overexpression through the IRF-1α sequence.

To determine the targets of EBNA1 in JAK2/STAT1/IRF-1 signaling proteins, changes in the expression of the signaling proteins in IFNγ-stimulated or unstimulated SNU-719 cells were measured upon EBNA1 knockdown. Regardless of IFNγ stimulation, EBNA1 knockdown reduced mRNA and protein levels of JAK2 and STAT1 in SNU-719 cells (Fig. 7C,D). IRF-1 and PD-L1 mRNA levels were also significantly decreased upon EBNA1 knockdown in IFNγ-stimulated or unstimulated SNU-719 cells (Fig. 7C). These results indicate that EBNA1 enhances the transcriptional expression of JAK2 and subsequently activates the constitutive and IFNγ-induced STAT1/IRF1/PD-L1 signaling in EBV (+) GC cells (Fig. 7E).

## Discussion

In this study, we revealed that constitutive PD-L1 expression is correlated with EBNA1 expression in EBV (+) GC cell lines without *PD-L1* gene amplification. Although IFNγ promotes PD-L1 overexpression in both EBV (−) and EBV (+) GC cells, notably, EBV (+) GC cells show significantly higher PD-L1 expression by activating JAK2/STAT1/IRF-1 signaling compared to EBV (−) GC cells. The IFNγ-induced IRF-1 was shown to bind to the IRF-1α DNA sequence of the *PD-L1* promoter in EBV (+) GC cells. EBNA1 knockdown reduces the constitutive and IFNγ-inducible *PD-L1* promoter activity by decreasing the transcript and protein levels of JAK2 and subsequently the STAT1/IRF-1/PD-L1 signaling.

This study found that the levels of EBNA1 expression are correlated with constitutive and inducible PD-L1 levels in the three EBV (+) GC cell lines. EBV (+) GC is known to express only two latent viral proteins, EBNA1 and LMP2A. Among these, EBNA1 is expressed in almost all patients with EBV (+) GC, whereas LMP2A is detected in approximately 50% of patients with EBV (+) GC^14,15^. EBNA1 is a viral nuclear protein essential for the maintenance of the EBV genome and has a *cis*-acting mechanism to prevent immune recognition of EBNA1 expressing tumor cells by cytotoxic T lymphocytes^23^. The size of EBNA1 protein in EBV (+) cell lines was dependent on the GAr size of EBNA1^18^. Interestingly, this study showed that the smaller the size of EBNA1, more specifically the GAr size, the higher the expression of EBNA1 and PD-L1. Differences in EBNA1 expression between EBV (+) GC cell lines do not appear to be due to differences in EBV copy number among EBV (+) GC cell lines. Despite SNU-719 and YCCEL1 cell lines have similar numbers of EBV copies per cell^24^, EBNA1 expression levels were different in this study. The GAr of EBNA1 suppresses the translation of EBNA1 mRNA, preventing the antigen peptide presentation to the MHC class I pathway, and eventually, play a role of immune evasion^18,25^. However, there has not been reported that the size of EBNA1 GAr is directly inversely proportional to the transcription and protein expression of EBNA1 mRNA and even to the expression level of PD-L1, rather than the translation of EBNA1 mRNA^25^. Thus, this direct association with the size of EBNA1 GAr and EBNA1 transcription requires further investigation. EBNA1 proteins of SNU-719 and YCCEL1 cells contain 589 and 625 amino acids, respectively. Compared to YCCEL1 cells, SNU-719 cells partially deleted the EBNA1 GAr and EBNA1 protein showed about 94.1% amino acid identity between two cell lines (Supplementary Fig. S3)^26^. The EBV genome of NCC-24 cell line has not yet been sequenced, but we found that the EBNA1 protein of NCC-24 is the shortest and the least expressed among the EBV (+) GC cell lines. Thus, our study indicates that EBNA1 may play a role in constitutive and IFNγ-inducible PD-L1 overexpression.

We found that IFNγ-induced PD-L1 overexpression is mediated by activation of JAK2/STAT1/IRF-1 signaling pathway in EBV (+) GC. Although IFNγ has been previously demonstrated to increase PD-L1 expression in GC cells^27^, the detailed mechanisms by which IFNγ induces PD-L1 expression in EBV (+) GC cells remain unclear. The GSEA using TCGA GC tissue data suggested that IFNγ could activate the JAK/STAT signaling pathway in EBV (+) GC. In addition, immunohistochemical analysis using EBV (+) GC tissues revealed that PD-L1 was strongly expressed throughout EBV (+) GC cells infiltrating with many T lymphocytes. Thus, it was hypothesized that IFNγ released from tumor-infiltrating T lymphocytes could activate the JAK/STAT signaling pathway in EBV (+) GC cells. In this study, we displayed that IFNγ stimulation promotes PD-L1 overexpression by activating JAK2/STAT1/IRF-1 signaling in EBV (+) GC. Furthermore, IFNγ-induced IRF-1 was found to bind to the IRF-1α DNA sequence of the *PD-L1* promoter. Activation of this PD-L1 signaling pathway was reaffirmed in EBV (+) GC because the IFNγ-stimulated JAK2/STAT1/IRF-1/PD-L1 signaling was suppressed with AZD1480 (an ATP-competitive JAK2 inhibitor) and fludarabine (a STAT1 inhibitor which causes a specific depletion of STAT1 protein and mRNA), respectively.

EBNA1 knockdown reduces the basal and inducible PD-L1 expression levels and decreased *PD-L1* promoter activity in EBV (+) GC cells. EBNA1 induces transcriptional upregulation of JAK2, leading to constitutive and IFNγ-induced PD-L1 expression. In contrast, LMP2A did not upregulate PD-L1 expression and *PD-L1* promoter activity in constitutive and IFNγ-induced EBV (+) GC cells, based on LMP2A knockdown experiments (Supplementary Fig. S4). However, consistent with previous reports^28,29^, the effect of suppressing endogenous EBNA1 expression by RNA interference was unsatisfactory because siRNA against EBNA1 did not silence EBNA1 expression in SNU-719 cells by more than 40%. It is well known that endogenous EBNA1 expression can be knocked down by more than 90% in HeLa cells, whereas endogenous EBNA1 expression by RNA interference can only be reduced by 29%–41% in SNU-719 cells^28^. Thus, some secondary structural features of EBNA1 mRNA or interaction with other proteins may interfere with siRNA binding, but other knockdown methods for EBNA1, such as the lentiviral or CRISPR-dead cas9-mediated suppression system, need to be attempted.

Under basal conditions, EBV (−) AGS cells already express high levels of PD-L1 protein, most likely due to an intrinsic oncogenic activation^30–33^. Unlike EBV (+) SNU-719 cells, IFNγ-stimulation on EBV (−) AGS cells seems to activate the STAT1/IRF-1/PD-L1 signaling without going through the phosphorylation step of JAK2. Moreover, when EBNAl-transfected AGS cells were stimulated with IFNγ, AGS cells appeared to have only the effect of IFNγ, but there was no effect of ectopic EBNAl expression (data not shown). Additional experiments such as ectopic EBNA1 expression in EBV (−) GC cell lines without PD-L1 expression, including MKN-1 and SNU-601 cells, are required.

In summary, our results indicate that IFNγ-mediated PD-L1 overexpression is regulated by the JAK2/STAT1/IRF-1 signaling pathway in EBV (+) GC cells. IRF-1 directly regulates *PD-L1* transcription by binding to the IRF-1α site of the *PD-L1* promoter. In addition, EBNA1 partially enhances IFNγ-mediated PD-L1 expression through upregulation and activation of JAK2. Recently, anti-PD-L1 and anti-PD-1 antibodies to the PD-L1/PD-1 axis have been shown to be encouraging as immunotherapies in a variety of cancers^34^. Given that PD-L1 is strongly expressed upon IFNγ stimulation in EBV (+) GC cells, the immune evasion of EBV (+) GC cells could be regulated by inhibiting JAK2/STAT1/IRF-1 signaling as well as by controlling EBNA1 expression.

## Materials and Methods

### Cell lines and tissue samples

The gastric cancer cell line AGS was obtained from the American Type Culture Collection (Manassas, VA, USA). EBV (−) GC cell lines (SNU-601, MKN-1, and MKN-28) and EBV (+) GC cell lines (SNU-719 and NCC-24) were obtained from the Korean Cell Line Bank (Seoul, Korea). The six cell lines were cultured in RPMI1640 medium. The EBV (+) YCCEL1 GC cell line was a kind gift from Dr. SY Rha from the Yonsei University College of Medicine. YCCEL1 cells were cultured in minimum essential medium (MEM). Media were supplemented with 10% fetal bovine serum (Hyclone, Logan, UT, USA) and 1% penicillin/streptomycin (Life Technologies, Carlsbad, CA, USA). Cultures were maintained at 37 °C in a humidified atmosphere containing 5% CO_2_. Informed consent was obtained from all patients and the experimental protocol was reviewed and approved by the Tissue Ethical Committee of Korea University Ansan Hospital for the use of tissue specimens (No. 2016-004). The method was carried out in accordance with the committee’s approved guidelines. Paraffin-embedded GC and non-malignant gastric tissue specimens were used for immunohistochemistry.

### Quantitative reverse transcription polymerase chain reaction (qRT-PCR)

Total RNA was extracted from cell lines using TRIzol (Invitrogen, Carlsbad, CA, USA) and subjected to reverse transcription. qRT-PCR was performed in triplicate using a 7500 Real-Time PCR System and Power SYBR^®^ Green Gene Expression Assays (Thermo Fisher Scientific). Primer sequences are summarized in Supplementary Table S1. *β*-actin was amplified in the same reaction to serve as an internal control for normalization. Fold changes in gene expression were measured using the comparative threshold cycle method (ΔΔCt).

### Western blotting and immunohistochemistry

Protein lysates were subjected to electrophoresis on 10% sodium dodecyl sulfate polyacrylamide gels and transferred to polyvinylidene difluoride membranes. Blots were incubated using primary anti-PD-L1, JAK2, pJAK2 (Y1007/1008), STAT1, pSTAT1 (Y701) (Cell Signaling Technology, Beverly, MA, USA), IRF-1, EBNA1, and *β*-actin (Santa Cruz Biotechnology, Dallas, TX, USA) antibodies. Blots were incubated with horseradish peroxidase-conjugated secondary antibodies (Cell Signaling Technology) and visualized using ECL (Amersham Biosciences, Buckinghamshire, UK). Immunohistochemical analysis was performed using the Leica BOND-MAX autostainer and Leica Refine detection kit (Leica Biosystems, Melbourne, Australia) using primary PD-L1 antibody (1:50, clone E-7, Santa Cruz Biotechnology).

### *PD-L1* copy number analysis

qRT-PCR for *PD-L1* DNA copy number was performed in triplicate using a 7500 Real-Time PCR System (Thermo Fisher Scientific) and TaqMan^®^ *PD-L1* Copy Number Assays (Hs03704252_cn, Thermo Fisher Scientific) with Reference Assay RNase P (Thermo Fisher Scientific). Data were analyzed using CopyCaller^®^ Software v2.0 (Thermo Fisher Scientific). Average value from five normal gastric tissues was used as a normal control.

### Dual luciferase reporter assay

The luciferase reporters of *PD-L1* promoter were constructed using pGL4.15 vector. The primer sequences are summarized in Supplementary Table S2. PCR products were cloned into the pGL4.15 vectors at the KpnI, SacI, and XhoI sites, which were verified by DNA sequencing. Cells were grown to 70% confluence for 24 h, serum-starved for 30 min, and transiently transfected with *PD-L1* promoter constructs using Lipofectamine 2000^®^ (Life Technologies). After 4 h, the culture medium was replaced with complete medium with or without IFNγ (R&D systems, Minneapolis, MN, USA). At 24 h after transfection, the cells were washed and lysed. Cell lysates were analyzed for luciferase activity using a dual luciferase assay kit (Promega) and an EnSpire multimode reader (PerkinElmer, Waltham, MA, USA). The *Renilla* luciferase construct, pRL-TK, was co-transfected as an internal control.

### Treatment with JAK2 and STAT1 inhibitors

AZD1480 and fludarabine were purchased from selleckchem (Houston, TX, USA). For the signaling inhibition experiments, AZD1480 and fludarabine dissolved in 100% dimethyl sulfoxide (DMSO, Sigma-Aldrich, Darmstadt, Germany) were prepared in a 10 mM stock and stored at −70 °C. SNU-719 cells were seeded in 6-well plates at a density of 1 × 10^6^ cells and treated with 5, 10, 20 μM of AZD1480 or fludarabine for 2 h, followed by treatment with 10 ng/mL IFNγ for 24 h. The cells harvested and analyzed for mRNA and protein expressions. DMSO was included in each plate as a negative control.

### Chromatin immunoprecipitation (ChIP) analysis

Physical associations between the IRF-1 and IRF-1 binding sites of the *PD-L1* promoter in SNU719 and AGS cells were analyzed using a ChIP assay kit (Millipore, Bedford, MA, USA). Cells were treated with formaldehyde for 10 min at 37 °C, incubated in lysis buffer, and sonicated to fragment the chromatin. The crosslinked protein-DNA complexes were immunoprecipitated with anti- IRF-1 antibody (Santa Cruz Biotechnology). Anti-histone 3 (Abcam, Cambridge, UK) antibody and Rabbit IgG (Cell Signaling Technology) were used as positive and negative controls, respectively. Cross-links were reversed after performing a pull-down assay on antibody-bound complexes with protein A-agarose/salmon sperm DNA (Millipore). DNA was recovered by phenol/chloroform extraction and ethanol precipitation and used as a template for subsequent PCR amplification. The primer sequences for PCR are summarized in Supplementary Table S3.

### Electrophoretic mobility shift assay (EMSA)

Nuclear extracts were obtained using a nuclear extract kit (Active Motif, Carlsbad, CA, USA) and were incubated with a biotin-labeled probe for 20 min in binding buffer (Active Motif). For supershift analysis, extracts were incubated with anti-IRF-1 antibody (Santa Cruz Biotechnology) and control antibody (Cell Signaling Technology). Reaction mixtures were separated on 6% acrylamide gels. Wild-type and mutant probe sequences are listed in Supplementary Table S4.

### *EBNA1* siRNA transfection

SNU-719 cells were grown to 70% confluence for 24 h, serum-starved for 30 min, and transiently transfected using Lipofectamine^®^ RNAiMAX (Thermo Fisher Scientific). The EBNA1 siRNA sequences are summarized in Supplementary Table S5. The siRNA (50 nM) and transfection reagent (9 μL) were each diluted with 250 μL of Opti-MEM^®^ medium (Thermo Fisher Scientific) and combined afterwards. The resulting mixtures were incubated for 5 min and added dropwise to each culture well containing 1 mL of Opti-MEM^®^ medium. After 4 h, the medium was replaced with the fresh complete RPMI1640 medium. Cells were cultivated for 48 or 72 h, washed in phosphate-buffered saline (PBS, Sigma-Aldrich), and harvested.

### Gene set enrichment analysis (GSEA)

GSEA was performed using Bioconductor R packages, including TCGAbiolinks and Ensemble of GSEA (EGSEA)^35,36^. RNA-sequencing data were downloaded and preprocessed using TCGAbiolinks and annotated with Entrez ID. Differentially expressed genes between EBV (+) and EBV (−) CIN-type GCs were identified using the limma-voom with adjustment for false discovery rate and hierarchical clustering was performed using the heatmap.2 function of the gplots. Enrichment for differential regulated gene sets was calculated using EGSEA. Hallmark gene sets were downloaded from the MSigDB database (http://software.broadinstitute.org/gsea/msigdb). Fold changes of gene expression were projected onto Kyoto Encyclopedia of Genes and Genomes (KEGG) maps (http://www.genome.jp/kegg/)^37^. OncoPrint analysis of GC TCGA was performed on the cBioPortal for Cancer Genomics.

### Statistical analysis

Experiments were repeated at least three times. Mean values were compared using two-tailed Student’s *t*-test and one-way analysis of variance (ANOVA). Statistical significance was considered at *p*-value < 0.05. All statistical analyses were performed with SPSS for Windows 10.0 (SPSS Inc, Chicago, IL, USA).

## Electronic supplementary material

Supplementary information
