# Supplementary material for: IFNγ induces PD-L1 overexpression by JAK2/STAT1/IRF-1 signaling in EBV-positive gastric carcinoma
Source: Sci Rep. 2017 Dec 19;7:17810. doi: 10.1038/s41598-017-18132-0 (PMC5736657; doi:10.1038/s41598-017-18132-0)
Supplement: Supplementary file 1 — Supplementary information [file 41598_2017_18132_MOESM1_ESM.doc]

**IFNγ induces PD-L1 overexpression by JAK2/STAT1/IRF-1 signaling in EBV-positive gastric carcinoma**

**Ji Wook Moon^1^, Su-Kang Kong^1^, Byung Soo Kim^1^, Hyun Ji Kim^1^, Hyangsoon Lim^1^, Kyeong A Noh^1^ , Younghye Kim^2^, Jung-Woo Choi^2^, Ju-Han Lee^2^ and Young-Sik Kim^1, 2^**

^1^Department of Pathology, Korea University College of Medicine, Seoul, Republic of Korea

^2^Department of Pathology, Korea University Ansan Hospital, Ansan, Republic of Korea

**Supplementary Table S1.** Primer sequences used for mRNA quantitation.

| **Genes** | **Primer sequences (5'-3')** | | **Amplicons**  **(bp)** | **Annealing**  **Temperature**  **(℃)** | **References** |
| --- | --- | --- | --- | --- | --- |
| IFNGR1 | F: | GTGTGAGCAGGGCTGAGAT | 109 | 60 | [1] |
|  | R: | TCCCAATATACGATAGGGTTCA |  |  |  |
| IFNGR2 | F: | TTAAATACACCGACAGTAAATGGT | 321 | 60 | [2] |
|  | R: | AAAGGCCGTGGAGGTATCAGCGATG |  |  |  |
| JAK2 | F: | TGCCGGTATGACCCTCTACA | 168 | 60 | NM_004972.3 |
|  | R: | ACCAGCACTGTAGCACACTC |  |  |  |
| STAT1 | F: | GTTATGGGACCGCACCTTCA | 128 | 60 | NM_007315.3 |
|  | R: | CAGTGAACTGGACCCCTGTC |  |  |  |
| IRF-1 | F: | AAAGTCGAAGTCCAGCCGAG | 103 | 60 | NM_002198.2 |
|  | R: | CAGAGTGGAGCTGCTGAGTC |  |  |  |
| EBNA1 | F: | CAAGGAGGTTCCAACCCGAA | 150 | 60 | AP015015.1 |
|  | R: | ATATACGAACACACCGGCGA |  |  |  |
| LMP2A | F: | TCCCTAGAAATGGTGCCAATG | 107 | 60 | [3] |
|  | R: | GAAGAGCCAGAAGCAGATGGAT |  |  |  |
| PD-L1 | F: | TATGGTGGTGCCGACTACAA | 157 | 60 | [4] |
|  | R: | TGCTTGTCCAGATGACTTCG |  |  |  |
| *β*-actin | F: | AGAGCTACGAGCTGCCTGAC | 184 | 60 | NM_001101.3 |
|  | R: | AGCACTGTGTTGGCGTACAG |  |  |  |

**Supplementary Table S2.** Primer sequences used for *PD-L1* Promoter constructs.

| **Promoter constructs** | **Primer sequences (5'-3')** | | **Location** | **Restriction**  **enzyme** |
| --- | --- | --- | --- | --- |
| Full promoter | F: | cgcGGTACCTATGGGTCTGCTGCTGACTTT | -456 to +151 | KpnI |
|  | R: | cccCTCGAGGCGAGCTAGCCAGAGATACTG |  | XhoI |
| IRF-1α deletion promoter | F: | cgcGGTACCTATGGGTCTGCTGCTGACTTT | -456 to -193 | KpnI |
|  | R: | gtacctGAGCTCAATGAGATTTTCACCGGGAA |  | SacI |
|  | F: | gtacctGAGCTCCGAAGGTCAGGAAAGTCCAA | -140 to +151 | SacI |
|  | R: | cccCTCGAGGCGAGCTAGCCAGAGATACTG |  | XhoI |
| IRF-1β deletion promoter | F: | cgcGGTACCTATGGGTCTGCTGCTGACTTT | -456 to -118 | KpnI |
|  | R: | gtacctGAGCTCTGTTGGTGTCCTAGGAATAAAGC |  | SacI |
|  | F: | gtacctGAGCTCCGAAGGTCAGGAAAGTCCAA | - 81 to +151 | SacI |
|  | R: | cccCTCGAGGCGAGCTAGCCAGAGATACTG |  | XhoI |
| IRF-1α,1β deletion promoter | F: | cgcGGTACCTATGGGTCTGCTGCTGACTTT | -456 to -193 | KpnI |
|  | R: | gtacctGAGCTCAATGAGATTTTCACCGGGAA |  | SacI |
|  | F: | gtacctGAGCTCCGAAGGTCAGGAAAGTCCAA | - 81 to +151 | SacI |
|  | R: | cccCTCGAGGCGAGCTAGCCAGAGATACTG |  | XhoI |

The lowercase and underlined nucleotides sequences are linker and restriction enzyme sites, respectively. The transcription start site is at +1 and the position of the promoter is indicated as location.

**Supplementary Table S3.** Primer sequences used for chromatin immunoprecipitation.

| **Genes** | **Primer sequences (5'-3')** | | **Amplicons**  **(bp)** | **Annealing**  **Temperature**  **(℃)** | **References** |
| --- | --- | --- | --- | --- | --- |
| IRF-1α | F: | TTCCCGGTGAAAATCTCATT | 95 | 60 | NC_000009.12 |
|  | R: | TGTTGGTGTCCTAGGAATAAAGC |  |  |  |
| IRF-1β | F: | GCTTTATTCCTAGGACACCAACA | 79 | 60 |  |
|  | R: | TTGGACTTTCCTGACCTTCG |  |  |  |

**Supplementary Table S4.** EMSA probes.

| Oligonucleotide | Primer sequence (5’ → 3’) | | Location |
| --- | --- | --- | --- |
| IRF-1α |  |  | -182 to -153 |
| Wild-type | F: | ACT GGA CTG ACA TGT TTC ACT TTC TGT TTC |
|  | R: | GAA ACA GAA AGT GAA ACA TGT CAG TCC AGT |
| Mutant | F: | ACT GGA CTG ACA TGT GTA ACC TTC TGT TTC |
|  | R: | GAA ACA GAA GGT TAC ACA TGT CAG TCC AGT |
|  |  |  |  |
| IRF-1β |  |  | -116 to -87 |
| Wild-type | F: | CTA GAT ACC TAA ACT GAA AGC TTC CGC CGA |
|  | R: | TCG GCG GAA GCT TTC AGT TTA GGT ATC TAG |
| Mutant | F: | CTA GAT ACC TAA GCT TAC AGC TTC CGC CGA |
|  | R: | TCG GCG GAA GCT GTA AGC TTA GGT ATC TAG |

The underlined nucleotide sequence is a mutated base^5^. The transcription start site is at +1 and the position of the promoter is indicated as location.

**Supplementary Table S5.** Primer sequences used for RNA interference.

| Gene | siRNA Primer sequence (5’ → 3’) | References |
| --- | --- | --- |
| *EBNA1* siRNA1 | Sense: GGAGGUUCCAACCCGAAAUdTdT | [6] |
|  | Anti-sense: AUUUCGGGUUGGAACCUCCdTdT |  |
| *EBNA1* siRNA2 | Sense: GUAAGACCUCCCUUUACAACCUCAGdTdT | [7] |
|  | Anti-sense: CUGAGGUUGUAAAGGGAGGUCUUACdTdT |  |
| *LMP2A* siRNA1 | Sense: AAUUCCCAAUACCCAUCUGCUdTdT | [3] |
|  | Anti-sense: AGCAGAUGGAUAUUGGGAGUUdTdT |  |
| Scramble siRNA | Sense: UUCUCCGAACGUGUCACGUdTdT | [8] |
|  | Anti-sense: ACGUGACACGUUCGGAGAAdTdT |  |


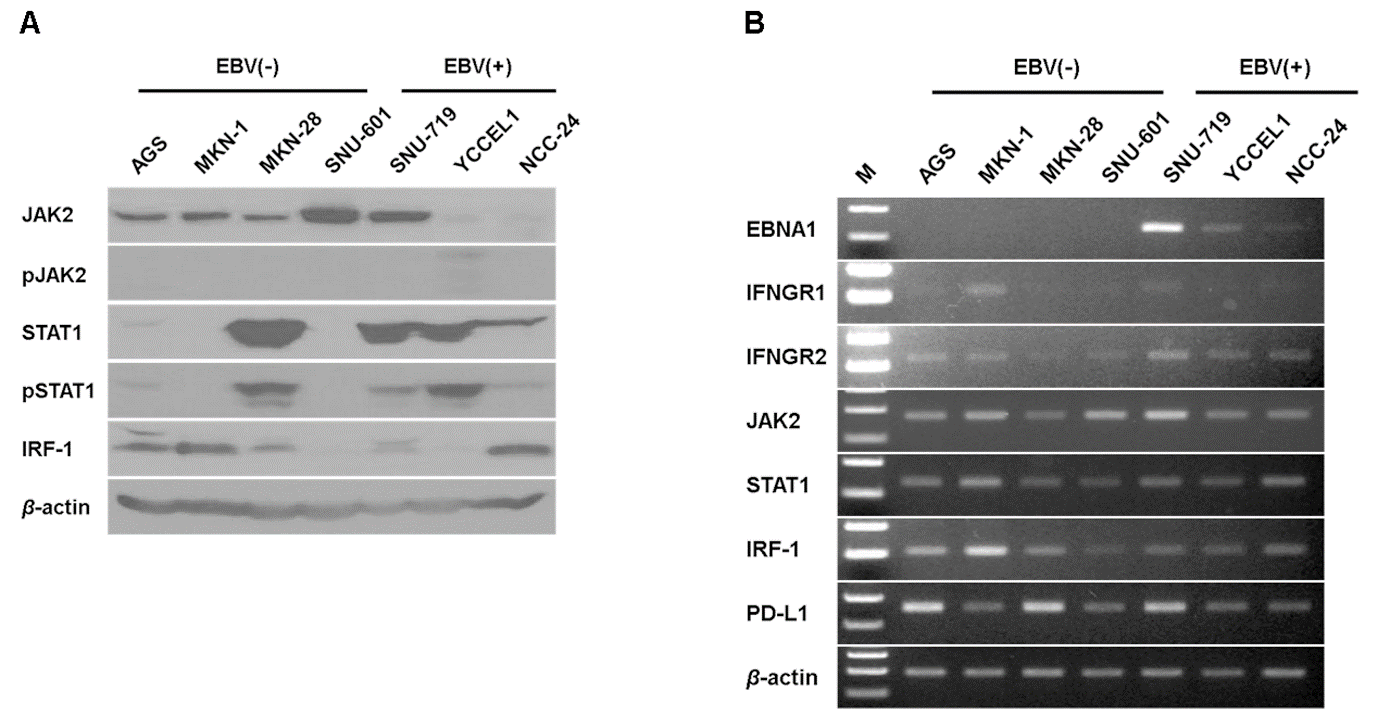


**Supplementary Figure 1.** Constitutive expression of JAK2/STAT1/IRF-1 signaling pathway. (A) Relative protein levels of JAK2, pJAK2, STAT1, pSTAT1, and IRF-1 were determined by immunoblot analysis. (B) Relative mRNA levels of EBNA1, IFNGR1, IFNGR2, JAK2, STAT1, IRF-1, and PD-L1 in seven GC cell lines were determined by RT-PCR. *β*-actin was used as a loading control. M, 100 bp DNA ladder.


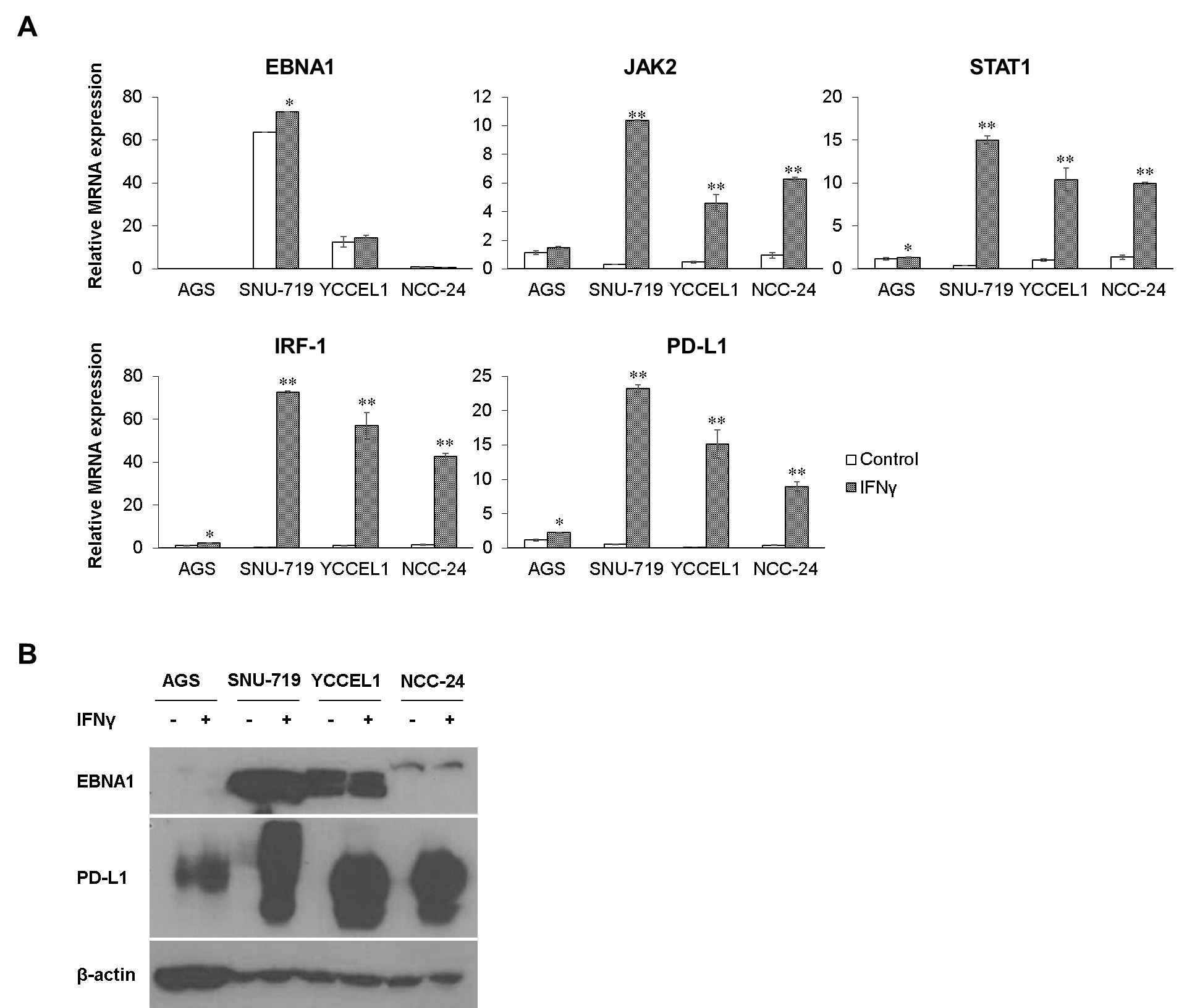


**Supplementary Figure 2. IFNγ induces significantly higher PD-L1 expression in three EBV (+) SNU-719, YCCEL, and NCC-24 cells compared to EBV (-) AGS cells. Inducible PD-L1 expression correlates with EBNA1 expression.** (A) After AGS, SNU-719, YCCEL1, and NCC-24 cell lines were stimulated with 10 ng/mL IFNγ for 24 h, relative mRNA expression of EBNA1, JAK2, STAT1, IRF-1, and PD-L1 compared to the unstimulated GC cells were determined by qRT-PCR. The data are presented as mean ± SEM (n=3). *P < 0.05 and *P < 0.001 compared to the same unstimulated cells (Student's *t*-test). (B) After AGS, SNU-719, YCCEL1, and NCC-24 cell lines were stimulated with 10 ng/mL IFNγ for 24 h, protein levels of EBNA1 and PD-L1 were compared with those of the same unstimulated cells by immunoblotting.


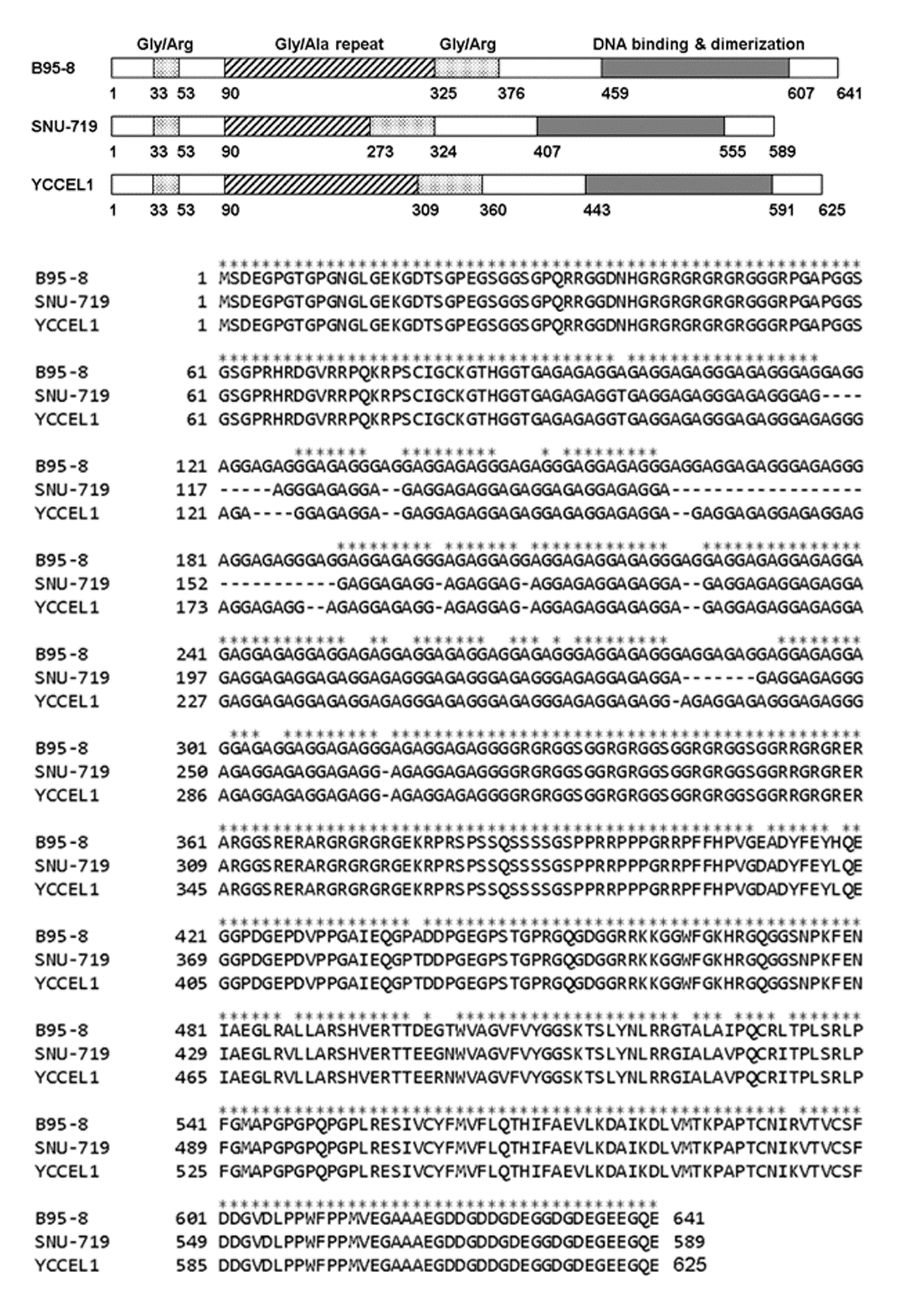


**Supplementary Figure 3. Schematic representation of EBNA1 protein in B95-8, SNU-719, and YCCEL1 cell lines.** In B95-8 (GenBank: V01555.2), SNU-719 (GenBank: AP015015.1) and YCCELl (GenBank: AP015016.1) cell lines, the EBNA1 proteins consist of 641, 589, and 625 amino acids, respectively. Amino acid sequences of EBNA1 from B95-8, SNU-719, and YCCEL1 cells are compared. * indicates that the same amino acid in EBNA1 is present in all three cell lines.


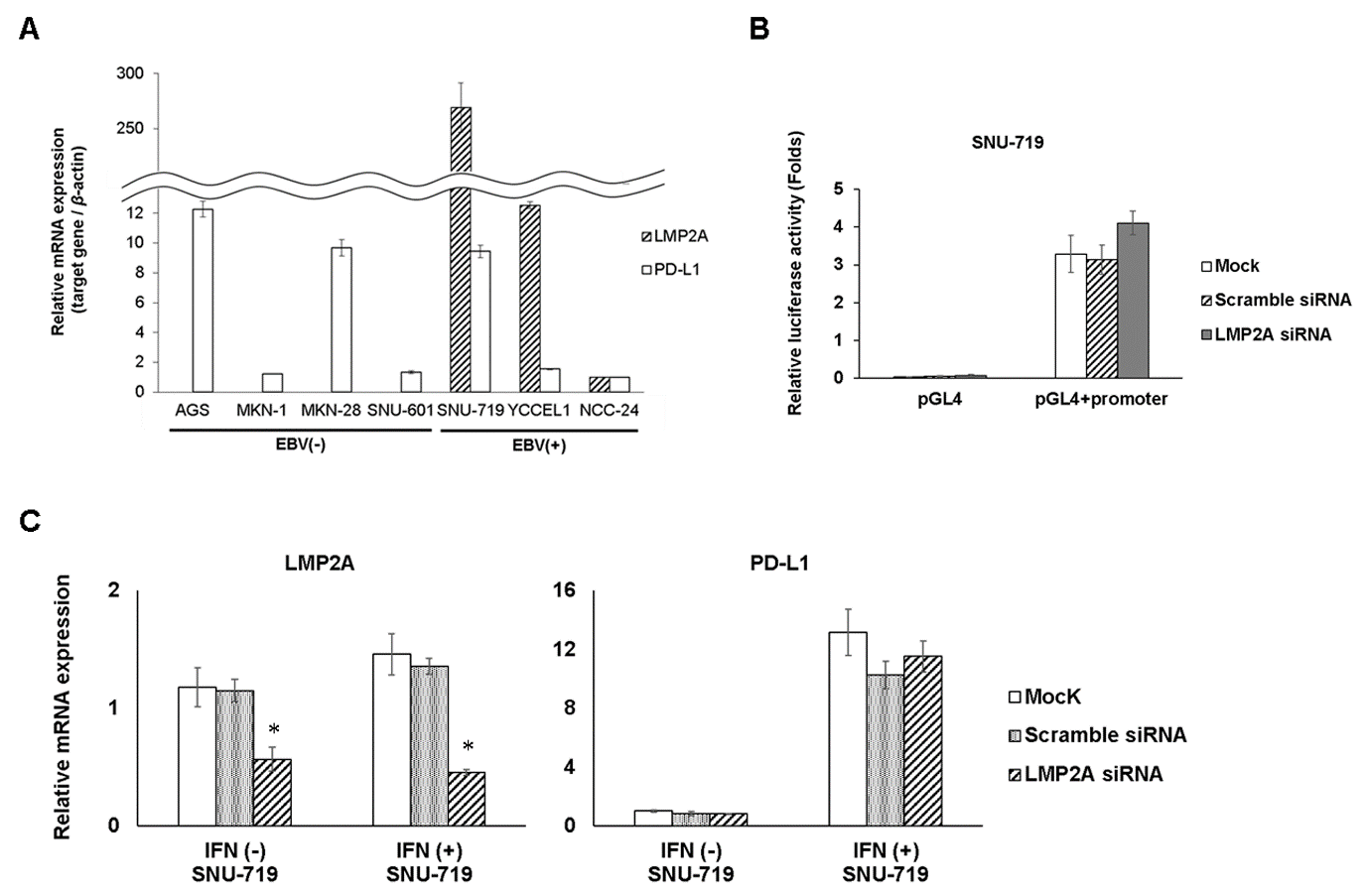


**Supplementary Figure 4.** PD-L1 expression is not associated with LMP2A expression in EBV (+) GC. (A) Relative mRNA expression levels of LMP2A and PD-L1 in seven GC cell lines were quantified by qRT-PCR. (B) After SNU-719 cells were transfected with LMP2A siRNA or scramble siRNA for 48 h, luciferase activities of the *PD-L1* promoter constructs were determined. (C) After SNU-719 cells were transfected with LMP2A siRNA or scramble RNA for 48 h, cells were stimulated with or without IFNγ for 24 h. Relative mRNA levels of LMP2A and PD-L1 were quantified by qRT-PCR. The data are presented as mean ± SEM (n=3). *P < 0.05 compared to the SNU-719 cells transfected with mock or scramble siRNA (Student's *t*-test).

**References**

1. Wee, Z.N. *et al*. EZH2-mediated inactivation of IFN-gamma-JAK-STAT1 signaling is an effective therapeutic target in MYC-driven prostate cancer. Cell Rep 8(1):204-216. doi: 10.1016/j.celrep.2014.05.045 (2014).

2. Groux, H, *et al*. Induction of human T helper cell type 1 differentiation results in loss of IFN-gamma receptor beta-chain expression. J Immunol 158(12):5627-5631 (1997).

3. Guasparri, I., Bubman, D., Cesarman, E. EBV LMP2A affects LMP1-mediated NF-kappaB signaling and survival of lymphoma cells by regulating TRAF2 expression. Blood 111(7):3813-3820. doi: 10.1182/blood-2007-03-080309 (2008).

4. Haile, S.T. *et al*. Tumor cell programmed death ligand 1-mediated T cell suppression is overcome by coexpression of CD80. J Immunol 186(12):6822-6829. doi: 10.4049/jimmunol.1003682 (2011).

5. Lee, S.J. *et al*. Interferon regulatory factor-1 is prerequisite to the constitutive expression and IFN-gamma-induced upregulation of B7-H1 (CD274). FEBS Lett 580(3):755-762. doi: 10.1016/j.febslet.2005.12.093 (2006).

6. Sivachandran, N. *et al*. Contributions of the Epstein-Barr virus EBNA1 protein to gastric carcinoma. J Virol 86(1):60-68. doi: 10.1128/JVI.05623-11. PMID: 22013060 (2012).

7. Oh, S.T., Kim, M., Lee, S.K. Maintenance of the viral episome is essential for the cell survival of an Epstein-Barr virus positive gastric carcinoma cell line. Arch Pharm Res 32(5):729-736. doi: 10.1007/s12272-009-1512-7 (2009).

8. Moon, J.W. *et al*. Alcohol induces cell proliferation via hypermethylation of ADHFE1 in colorectal cancer cells. BMC Cancer 14:377. doi: 10.1186/1471-2407-14-377 (2014).
